# Connecting caddisworm silk structure and mechanical properties: combined infrared spectroscopy and mechanical analysis

**DOI:** 10.1098/rsob.160067

**Published:** 2016-06-08

**Authors:** Nicholas N. Ashton, Huaizhong Pan, Russell J. Stewart

**Affiliations:** Department of Bioengineering, University of Utah, Salt Lake City, Utah 84112, USA

**Keywords:** silk, bioadhesive, Ca^2+^-phosphoserine complexes, caddisfly

## Abstract

The underwater silk of an aquatic casemaking caddisfly larvae (*Hesperophylax occidentalis)* is viscoelastic, and displays distinct yield behaviour, large strain cycle hysteresis and near complete recovery of its initial strength and stiffness when unloaded. Yield followed by a stress plateau has been attributed to sequential rupture of serial Ca^2+^-cross-linked phosphoserine (pS) β-domains. Spontaneous recovery has been attributed to refolding of the Ca^2+^/pS domains powered by an elastic network. In this study, native Ca^2+^ ions were exchanged with other metal ions, followed by combined mechanical and FTIR analysis to probe the contribution of pS/metal ion complexes to silk mechanical properties. After exchange of Ca^2+^ with Na^+^, the fibres are soft elastomers and the infrared spectra are consistent with C_v3_ symmetry of the –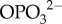 groups. Multivalent metal ions decreased the –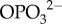 symmetry and the symmetric stretching modes (*v*_s_) split in a manner characteristic of ordered phosphate compounds, such as phosphate minerals and lamellar bilayers of phosphatidic acid lipids. Integrated intensities of the *v*_s_ bands, indicative of the metal ion's effect on transition dipole moment of the P–O bonds, and thereby the strength of the phosphate metal complex, increased in the order: Na^+^ < Mg^2+^ < Sr^2+^ < Ba^2+^ < Ca^2+^ < Eu^3+^ < La^3+^ < Zn^2+^ < Fe^2+^. With a subset of the metal ion series, the initial stiffness and yield stress of metal ion-exchanged fibres increased in the same order: 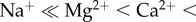 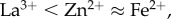 establishing the link between phosphate transition dipole moments and silk fibre strength.

## 1. Introduction

Aquatic caddisfly larvae (order Trichoptera) use sticky silk fibres like adhesive tape to construct protective shelters with species-specific designs using species-specific materials [1]. Casemakers, such as the species used in this study (*Hesperophylax occidentalis),* tape together adventitiously gathered stones into transportable body armour. The silk comprises a thin adhesive coating on a tough nanofibrous core. The adhesive coating contains glycoproteins and a peroxidase enzyme, which can catalyze covalent cross-linking to natural surface-active polyphenolic compounds as part of the adhesion mechanism [2]. The fibrous core is stiff (80–200 MPa) at low strains but dramatically softens, or yields, at 2–4% elongation, after which the stress plateaus. The fibres stiffen again before fracture at 100%–120% elongation. The work of elongation to fracture of approximately 17.3 ± 6.2 MJ m^−3^ is impressive for a fully hydrated biphasic material; the silk is tougher than tendon collagen [3], articular cartilage and the best synthetic double network hydrogels [4,5]. The abrupt softening at low strains is transient; silk fibres cyclically strained between 0% and 20% elongation fully and repeatedly recover their initial stiffness and yield stress within 120 min after unloading [6]. Significant for the caddisworm's lifestyle, the mechanical yield and stress plateau of the fibres shields the adhesive joints between fibre and substrate from irreversible damage, and repeated dissipation of strain energy without degradation of the silk's mechanical properties maintains the integrity of the composite case in its turbulent mountain stream environment.

The molecular basis for reversible yield of the silk fibres has been attributed to the sequential viscous unravelling of a serial network of Ca^2+^-cross-linked phosphoserine (pS) domains [6,7]. When unloaded, the mechanically denatured domains refold, guided by the memory of an elastic covalent network, resulting in nearly complete spontaneous recovery of the fibre's initial stiffness and strength [6]. The major structural protein of the fibre core by molecular mass (greater than 300 kg mol^−1^) and total mass, H-fibroin, comprises three imperfectly alternating repeats, which contain at least one (pSX)*_n_* motif, where pS is phosphoserine, X is usually an aliphatic residue or arginine and *n* is usually 4 or 5 [6,8]. In total, casemaker H-fibroins contain approximately 13 mol% pS residues. The occurrence of phosphorylated H-fibroin serines in all three caddisfly suborders points to the importance of the modification to caddisworm underwater silk structure and function throughout the order [8–10]. The proposal that H-fibroin (pSX)*_n_* motifs form a network of Ca^2+^-stabilized β-domains as the principal toughening mechanism of caddisworm silk is supported by multiple lines of experimental evidence. First, infrared (IR) and NMR spectroscopy, as well as X-ray scattering studies, showed decreased β-structure when Ca^2+^ was exchanged with Na^+^ [7,11,12]. Second, Ca^2+^-depleted silk fibres behaved mechanically like weak elastomers, with the initial stiffness decreased to 1% of native silk fibres, with no yield-like strain softening and no strain cycle hysteresis [7]. Third, a sharp pH-dependent decrease in fibre stiffness and strength coincided with protonation of H-fibroin phosphate groups as measured by IR spectroscopy [6].

Here, we describe experiments intended to further probe the relationship between the molecular structure and viscoelastic properties of caddisworm silk wherein native Ca^2+^ ions were exchanged with Na^+^, Mg^2+^, Zn^2+^, Fe^2+^, Eu^3+^, La^3+^, Sr^2+^ and Ba^2+^ ions. The stress response to controlled strains of fibres exchanged with a subset of these metal ions were correlated by Fourier transform infrared (FTIR) spectroscopy with effects on peptidyl-phosphate vibrational stretching modes, including frequency shifts, band splitting and integrated band intensities.

## 2. Material and methods

### 2.1. Silk harvesting

Fifth instar larvae of the casemaker *H. occidentalis* were collected just prior to pupation in the early summer in upper Red Butte Creek (Salt Lake County, UT, USA). Larvae were maintained in the laboratory and silk fibres were harvested from Teflon blocks as previously described [10].

### 2.2. Peptide synthesis

A (pSX)_4_ sequence (VpSIpSRpSVpSI) from the H-fibroin D-repeat was synthesized by solid phase peptide synthesis using standard Fmoc chemistry on 2-chlorotrityl chloride resin [13]. HBTU (2-(1*H*-benzotriazol-1-yl)-1,1,3,3-tetramethyluronium hexafluorophosphate) was used as the coupling agent and 20% piperidine in dimethylformamide (DMF) as the deprotection agent for Fmoc protected amino acids. Trifluoroacetic acid (TFA), triisopropylsilane (TIS), piperidine and DMF were from Sigma-Aldrich (Milwaukee, WI). *N*-α-Fmoc protected amino acids (Fmoc-AA-OH), *N*-α-Fmoc-*O*-benzyl-l-phosphoserine (Fmoc-Ser(PO(OBzl)OH)-OH) and 2-Cl-trityl chloride resin (100–200 mesh, 1.27 mmol g^−1^) were from EMD Biosciences (San Diego, CA). HBTU was from Advanced ChemTech (Louisville, KY). The peptide was cleaved from the resin with TFA/H_2_O/TIS (95/2.5/2.5) for 2 h. Yield was 150 mg (81%). The mass of the peptide was 659.7 g mol^−1^. A five wt% solution of the peptide in DI-H_2_O was adjusted to pH 9.0 with 6 M NaOH and lyophilized to obtain the Na^+^ salt of the synthetic peptide. The Ca^2+^ salt was prepared by adding CaCl_2_ to precipitate the synthetic peptide, which was then lyophilized.

### 2.3. Metal ion exchange

To exchange native Ca^2+^ with other metal ions, clean silk fibres were submerged for 1 h at ambient temperature in excess volumes of 10 mM solutions of the chloride salts of Mg^2+^, Zn^2+^, Fe^2+^, Eu^3+^, La^3+^, Sr^2+^ and Ba^2+^ buffered with 1 mM Bis-Tris and adjusted to pH 7.0 with NaOH. Multivalent metal ion exchange was facilitated by including 50 mM NaCl in the exchange solutions. The exchanged fibres were then equilibrated for 1 h in excess solutions containing 1 mM of the test metal chloride, plus 1 mM NaCl, 1 mM Bis-Tris and adjusted to pH 7.0 with NaOH. Fibres exchanged with Na^+^ were incubated for 1 h in 10 mM Na^+^-EDTA, 50 mM NaCl and 1 mM Bis-Tris (pH 7.0), then equilibrated in 1 mM NaCl, 1 mM EDTA and 1 mM Bis-Tris (pH 7.0). For elemental and attenuated total reflectance (ATR)-FTIR analysis, bundles of fibres were exchanged in bulk. For mechanical testing, metal ions were exchanged after single fibres were mounted in a submersion chamber on the material test system.

### 2.4. Elemental analysis by ICP-OES

Bundles of fibres were rinsed thoroughly with DI-H_2_O after metal ion exchange, lyophilized, dissolved in 40% nitric acid, analysed in triplicate by inductively coupled plasma optical emission spectrometry (ICP-OES) and the metal ions and P quantified by comparison to commercial standards (PerkinElmer, Optima 3100XL). Metal ion concentrations are reported as molar ratios to P to normalize the mass of fibres in each sample. After normalization, the propagated error is given by the fractional total deviation.

### 2.5. Mechanical testing

Isolated single silk fibres, 3–4 mm in length, were mounted on the fixtures of a custom micromaterials test system with Loctite 3301 UV cure adhesive, then submerged in an immersion chamber [6,7]. As described earlier, metal ion exchange for mechanical testing was done after single silk fibres were mounted in the immersion chamber. During mechanical tests, fibres were submerged in excess solutions of 1 mM of the test metal chloride or 1 mM Na^+^-EDTA. The test solutions contained 1 mM NaCl and 1 mM Bis-Tris and were adjusted to pH 7.0 with NaOH. The initial length of each mounted fibre was determined by pulling it taut until a measureable force was detected. The wide diameter of 10 fibres from each of the two larvae used in the study were optically measured under 100× magnification and used to approximate the cross-sectional area of the paired-fibre silk ribbons as 15.5 ± 1.4 and 13.6 ± 1.4 µm, for larva 1 and 2, respectively. Engineering stress was calculated assuming that the silk ribbons comprised two identical paired cylindrical sub-fibres. For all experiments the strain rate was 0.03 s^−1^. The maximum reliable acceleration/deceleration for the actuator motor, 5 mm s^−2^, was used. Elongation was initiated while the fibres were slack so that constant velocity was reached before the fibres were loaded. The initial silk fibre modulus, pseudo-yield stress, cycle hysteresis and toughness were computed from the force-elongation profiles with Matlab (MathWorks) software as previously described [6].

### 2.6. Single fibres strained to fracture

For each metal ion tested, five fibres were strained until complete rupture while submerged in the 1 mM metal ion test solutions described earlier (pH 7.0). The average of the five force–elongation profiles was plotted as a solid line with the standard deviation represented by shading. Mechanical tests were reported as force rather than engineering stress because Na^+^-exchanged silk fibres swelled fourfold in cross-sectional area [7]. Although the volume of the biphasic fibres changed, the number of covalent load-bearing cross-links did not change and it was therefore not appropriate to divide the force by the cross-sectional area.

### 2.7. Cyclical strains

A single silk fibre was first conditioned by cycling to 20% elongation and back in 1 mM CaCl, 1 mM NaCl and 1 mM Bis-Tris (pH 7.0) to replicate the natural hard water habitat of caddisworms. After a 2 h recovery period, the initial length of the fibre was re-measured. After the conditioning cycle, the initial length changed by 0.4% elongation. When conditioned fibres were subsequently cycled four times to 20% elongation with a 2 h rest between each cycle, there were negligible changes in the initial modulus, yield stress and cycle hysteresis between progressive cycles. Therefore, all fibres were first conditioned by cycling to 20% elongation in 1 mM CaCl solution, allowed to fully recover for 2 h and the initial length was re-measured.

To determine the effects of metal ion exchange on fibre mechanical properties, conditioned fibres were first cycled to 20% elongation one time in 1 mM CaCl_2_, 1 mM NaCl and 1 mM Bis-Tris (pH 7.0) solution to establish a mechanical baseline. Subsequent measurements of the mechanical parameters—initial stiffness, yield stress, cycle hysteresis and maximum stress at 20% elongation—after metal ion exchange are reported as percentage changes from the Ca^2+^ baseline mechanical parameters for that individual fibre. Mechanical tests of ion-exchanged silk fibres were performed while the fibres were submerged in the 1 mM metal chloride test solutions buffered with Bis-Tris at pH 7.0. Mechanical measurements were repeated with three fibres for each metal ion species tested. To simplify the visual representation of the 15 different fibres tested, the area within the control cycle hysteresis loops was normalized to the average hysteresis loop for all 15 control cycles. The normalized exchange cycles were then averaged for each ion species (*n* = 3) and plotted ± s.d.

### 2.8. ATR-FTIR spectroscopy of silk phosphoserines

The effect of metal ion exchange on the dianionic phosphate symmetric (*v*_s_) and (*v*_a_) and asymmetric stretching modes, which occur between 914–1186 cm^−1^, were monitored by ATR-FTIR. Metal ion-exchanged fibre bundles were clamped to the ATR crystal and excess solution was applied around the clamp to prevent dehydration. Multivalent metal ion-exchanged fibres were buffered at pH 7.0 with 1 mM Bis-Tris. At this pH the phosphates were fully deprotonated because monoanionic stretching modes were absent. Na^+^ exchanged fibres were fully deprontonated at pH 9.0 (1 mM Tris-HCl).

ATR-FTIR absorbance spectra were collected using a Nicolet 6700 spectrometer (Thermo Scientific, FL) with a diamond Smart iTR accessory, a deuterated triglycine sulfate detector and a KBr/Ge mid-infrared optimized beamsplitter. All spectra were recorded with a resolution of 4 cm^−1^ and 512 averaged scans. The silk spectra were corrected for water absorbance contributions and normalized using the area of the spectral region from 1428–1475 cm^−1^ containing absorption bands of pH insensitive side groups including Trp (CH) bending, Trp (CC) stretching, Trp (CN) stretching and CH_3_ bending mods of aliphatic sidechains like Leu, Ile, Val, Ala, Thr and Met using previously described methods [6]. The spectral region containing the serine phosphate symmetric and asymmetric absorbance modes, 914–1186 cm^−1^, was deconvolved with Gaussian curves using PeakFit software (Systat Software Inc.). A linear baseline with a 3% tolerance was applied and local minima in the second derivative were used to place the initial Gaussian constituents. The peak position, height and width were allowed to vary during the iterative least-square method employed in the PeakFit software thereby minimizing the residuals between the fitted and experimental spectra. The Na^+^ and Ca^2+^ phosphoserine salt spectra were adjusted with a linear baseline and the two peak areas from 924–1211 cm^−1^ were normalized to the average.

## 3. Results

### 3.1. Metal ion exchange

Silk fibres isolated from natural water contain multivalent metal ions in an approximately 1 : 1 molar ratio to phosphate groups [7,8]. Ca^2+^ is most abundant, followed by Mg^2+^, with much smaller ratios of Fe^2+^ and Zn^2+^. To exchange native metal ions with other multivalent ions, the fibres were incubated in a relatively high concentration, 10 mM, of the exchange ion. Inclusion of 50 mM NaCl in the exchange solution facilitated exchange. After metal ion exchange, the metal ion to P molar ratio was nearly 1 : 1 for Mg and Ca, higher for Zn and substantially higher for Fe (table 1). The high Fe/P ratios may have been due to iron hydroxide precipitation at pH 7.0 and entrapment within the fibres. To exchange the high affinity multivalent ions with low affinity monovalent Na^+^, the fibres were incubated with the chelator sodium EDTA to compete with the high affinity phosphate binding sites for multivalent ions.

**Table 1.** Metal to phosphorus molar ratios. Averages of three independent samples (mean ± fractional total deviation). Shaded values highlight the concentration of the intended exchange ion.

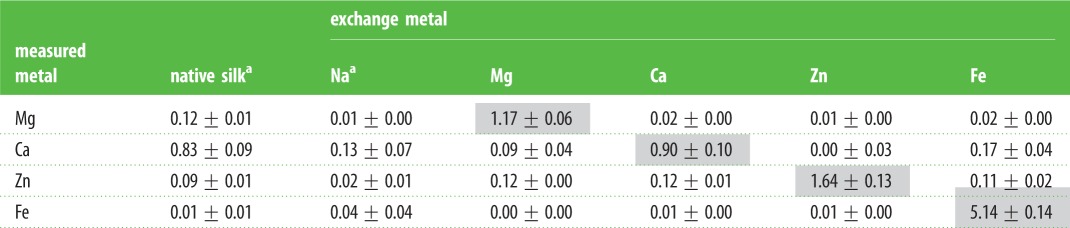

^a^Data from Ashton *et al*. [6].

### 3.2. IR spectroscopy of silk phosphoserine/metal ion complexes

The region of the IR spectrum from 800–1200 cm^−1^ contains absorption bands due to stretching modes of the P–O bonds of phosphate [14]. The number of IR-active P–O stretching vibrations is determined by the molecular symmetry of the phosphate group [15]. Changes in the number of bands upon metal complexation is indicative of changes in molecular symmetry and therefore provides information on the coordination environment of phosphate groups [16,17]. The frequencies and intensities of the vibrational bands are sensitive to bonding interactions, such as (de)protonation, H-bonding and complexation with metal ions. Coupling of vibrational modes in periodic structures—factor group splitting—can change the number, frequency and intensity of the bands [18].

**Figure 1.**
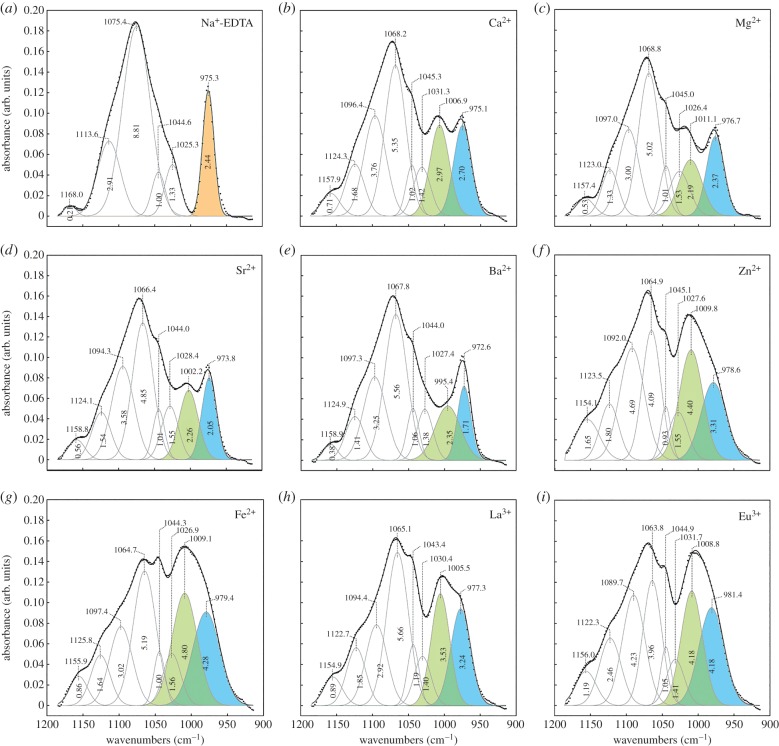
Effects of metal ions on peptidyl phosphate stretching modes. (*a*–*i*) Gaussian-fitted spectra of *Hesperophylax occidentalis* silk exchanged with the indicated metal ion and analysed in the hydrated state by ATR-FTIR. The split dianionic phosphate *v*_s_ bands, *v*_s1_ and *v*_s2_, are shaded blue and green, respectively, for multivalent metal ions. All spectra were collected at pH 7.0 except (*a*), which was collected at pH 9.0.

Fully deprotonated, the pyramidal 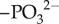 moiety of phosphoserine, with three resonance hybrid P–O bonds, has C_v3_ symmetry with two IR-active stretching vibrations, a P–O symmetric stretch and a P–O doubly degenerate asymmetric stretch [14]. A normalized and background subtracted AT-FTIR spectrum from 900–1200 cm^−1^ of hydrated silk fibres depleted of multivalent metal ions with Na^+^ EDTA, at pH 9, is shown in figure 1*a*. Consistent with C_v3_ symmetry, the 1075 cm^−1^ band was assigned to the degenerate asymmetric stretch (*v*_as_) and the 975 cm^−1^ band was assigned to symmetric stretch (*v*_s_) [19]. The weaker bands at 1168, 1114, 1045 and 1025 cm^−1^ were not assigned.

**Figure 2.**
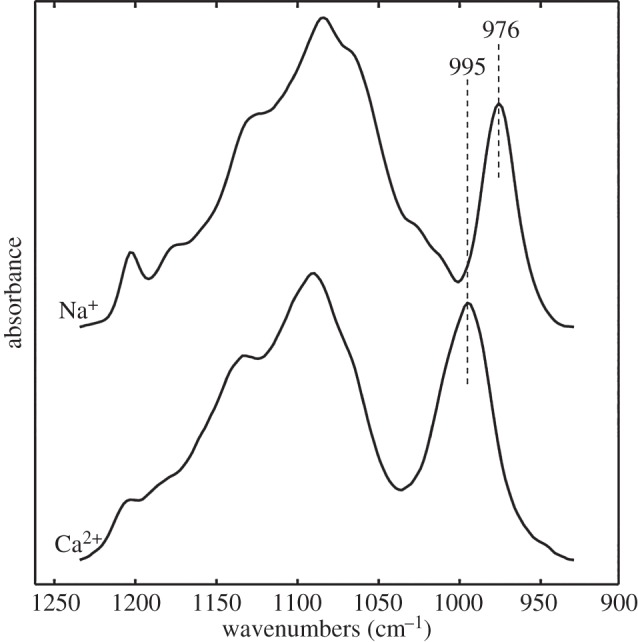
Phosphate IR spectra of Na^+^ and Ca^2+^ salts of synthetic (pSX)_4_ peptide. The dianionic phosphate *v*_s_ band shifted to higher frequency in the Ca^2+^ salt relative to the Na^+^ salt.

In Ca^2+^-containing fibres, the frequency of the 975 cm^−1^ *v*_s_ band was unchanged and its integrated intensity increased (figure 1*b*). Two bands at 1068 and 1096 cm^−1^, referred to as *v*_as1_ and *v*_as2_, appeared in place of the 1075 cm^−1^ *v*_as_ band. A new band appeared at 1007 cm^−1^ with similar integrated intensity as the 975 cm^−1^ *v*_s_ band. The appearance of two asymmetric stretching modes was evidence that the –PO_3_ symmetry of the phosphoserines was decreased to C*_v_*_2_ or lower by Ca^2+^ complexation. The similarity of the 1007 and 975 cm^−1^ bands to phosphate compounds with periodic structures, discussed later, suggested that the 1007 cm^−1^ band may have arisen from factor group splitting; the bands are therefore referred to as *v*_s1_ and *v*_s2_. A phosphopeptide (VpSIpSRpSVpSI) corresponding to a (pSX)_4_ motif from H-fibroin was synthesized to investigate whether the unique IR spectra of caddisworm silk was due to local sequence effects, e.g. alternating phosphoserines. Similar to other phosphoproteins, the v_s_ band shifted to 995 from 976 cm^−1^ in Ca^2+^ versus Na^+^ without the appearance of new bands (figure 2).

**Figure 3.**
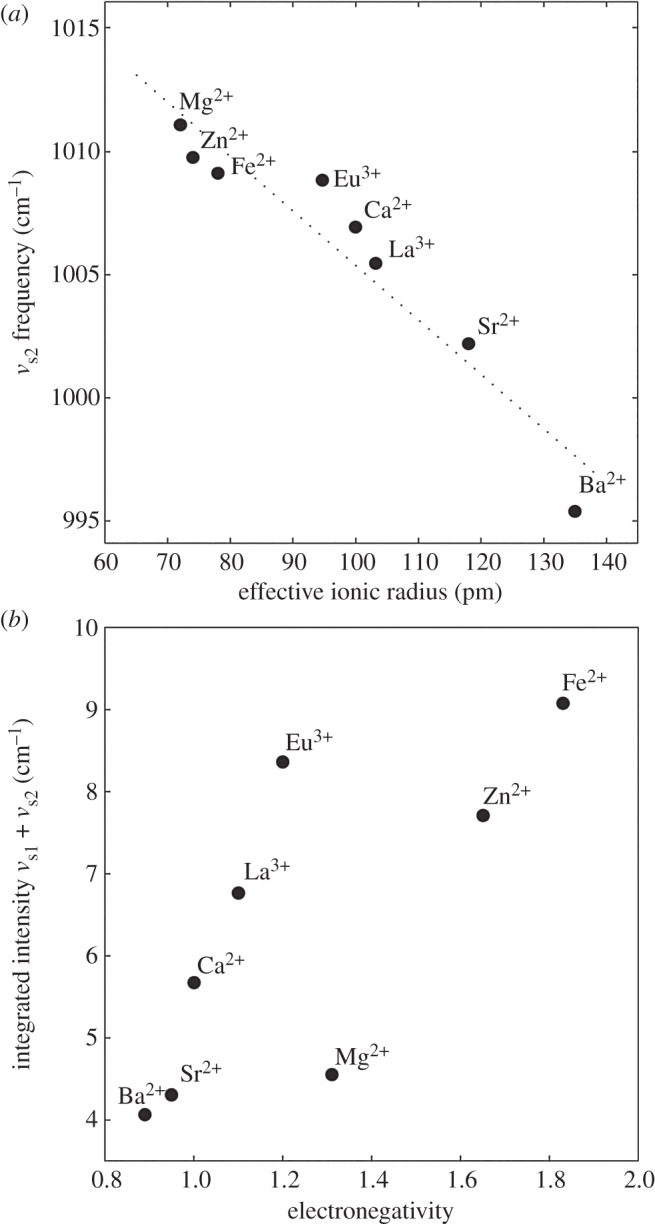
Dianionic phosphate *v*_s_ mode frequency and integrated intensity versus metal ion properties. (*a*) Ionic radius versus the *v*_s2_ band frequency. (*b*) Electronegativity versus the integrated area under the *v*_s1_ and *v*_s2_ bands.

ATR-FTIR spectra of fibres exchanged with Mg^2+^, Sr^2+^, Ba^2+^, Zn^2+^, Fe^2+^, La^3+^ and Eu^3+^ were qualitatively similar to Ca^2+^ fibres with regard to the number of IR bands, and therefore their qualitative effects on the symmetry of the 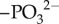 group were similar (figure 1*c*–*i*). The frequency of the *v*_s2_ band had a nearly linear dependence on the ionic radius of the multivalent metal ions (figure 3*a*) [20]. By contrast, the integrated intensity of the combined *v*_s1_ and *v*_s2_ bands was not dependent on ionic radius, but rather depended on the electronegativity of the multivalent metal ions, with the exceptions of Mg^2+^ and Zn^2+^ (figure 3*b*).

ATR-FTIR spectra from 900–1800 cm^−1^ of the metal ion-exchanged fibres (electronic supplementary material, figure S1) include the amide I region, which provides information on protein secondary structure. There was no discernible difference between the multivalent metal ions in the amide I region of the normalized and water-corrected spectra. We conclude there was not a significant effect of the tested multivalent metal ions on caddisworm silk β-sheet content. The effect on secondary structure as discerned by deconvolution of the amide I region when Ca^2+^ is exchanged with Na^+^ was previously published [6].

### 3.3. Mechanical assay of metal ion exchange effects on silk fibre structure

**Figure 4.**
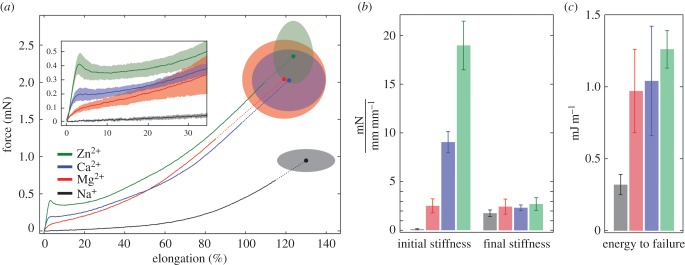
Metal ion-exchanged single silk fibres strained to fracture. (*a*) The average force–elongation profiles of fibres exchanged with the indicated metal ion then pulled to fracture (solid lines). The average fracture forces and strains are indicated by the symbol and ±1 s.d. by the shaded ellipses. *n* = 5 for each metal ion. Shading in the inset shows the standard deviations in the pseudo-yield region from 0% to 35% elongation. (*b*) The initial pre-yield stiffness and final stiffness just before failure are represented in units of force over strain. (*c*) The total energy absorbed before failure is reported per unit length of the fibre. In (*b*) and (*c*) the colours are the same as in panel (*a*).

Strong differential effects of multivalent metal ions on silk fibre mechanical properties were apparent in comparisons of the force–elongation profiles of silk fibres exchanged with Na^+^ to fibres exchanged with divalent metal ions (figure 4*a*). All mechanical tests were done at pH 7.0. The average initial stiffness of the Na^+^-exchanged fibres, 0.09 mN, was 1% of the stiffness of native and Ca^2+^-exchanged fibres and 0.5% of the stiffest Zn^2+^-exchanged fibres (figure 4*b*). The yield behaviour, interpreted molecularly as cooperative unfolding of serial H-fibroin domains comprising metal/phosphate complexes [6,7], was completely eliminated by depletion of multivalent cations. The Na^+^-exchanged fibres progressively strain stiffened toward the end of elongation before fracture (figure 4*b*) similar to elastomeric rubbers [21], and broke at slightly higher average elongations, 130 ± 14%, than divalent metal ion fibres, 119–124 ± 10–21% (figure 4*a*). Removing divalent metal ions revealed the covalent elastic network that provides a restoring force for spontaneous fibre recovery [4].

Comparing divalent metal ions to one another, the initial stiffness, yield force, and work to fracture (toughness) increased in the order of the expected stability of the metal ion/phosphates complexes: Mg^2+^ < Ca^2+^ < Zn^2+^ [3,22]. The average initial stiffness increased 660%, from 2.5 to 9.0 to 19.0 mN, the average yield force increased 430%, from 0.08 to 0.19 to 0.40 mN and the average work to fracture increased only 29.8%, from 0.97 to 1.04 to 1.26 mJ m^−1^ over the series (figure 4*a*–*c*). Work to fracture is presented for fibre length rather than fibre volume because the biophasic fibre cross-sectional area increases fourfold when divalent ions are exchanged with Na^+^ [7]. The total amount of material and the number of load-bearing covalent bonds is unchanged in the Na^+^ silk fibres swollen by an increase in the water phase. Beyond the yield point and force plateau, the divalent metal ion species had insignificant effects on the shape of the force–elongation profile. All three divalent cations displayed a similar magnitude of strain hardening before fracture, elongation at fracture, and fracture force (figure 4*a*,*b*). The final fibre stiffness was nearly independent of the metal ion, including Na^+^, which demonstrated that the divalent metal/phosphate stabilized domains were completely unfolded toward the end of the force–elongation profile. The increasing work to fracture (toughness) over the divalent metal ion series (figure 4*c*) was due almost entirely to the pre-yield region of the force–elongation curves, which demonstrated the importance of the Ca^2+^/phosphate domain structure on fibre toughness.

### 3.4. Cyclical strains

**Figure 5.**
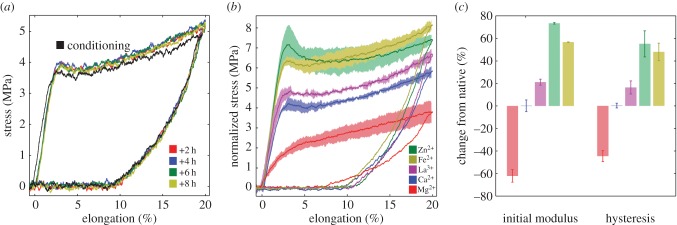
Cyclical strains of native and of ion-exchanged single silk fibres. (*a*) After the first conditioning cycle to 20% of a native fibre (black curve), subsequent cycles were superimposable on one another (coloured). The fibres were rested, unstrained, for 2 h between cycles. (*b*) The average cyclical force–elongation profile of three fibres exchanged with the indicated ion (solid lines). The shaded bands represent the s.d. (*n* = 3). (*c*) The percentage change in initial modulus and hysteresis from the control cycle values. Error bars represent the s.d. (*n* = 3).

Single native fibres were strained to 20% elongation at 2 h intervals to evaluate recovery of the fibre's initial mechanical properties after repeated strains (figure 5*a*). At the beginning of the second cycle, the fibre's unstrained initial length decreased by only 0.4% compared to the initial length at the beginning of the first cycle. After the first cycle, the initial stiffness and yield stress increased by approximately 7%. The second cycle and all subsequent cycles were nearly superimposable, demonstrating highly efficient recovery of the fibres from pseudo-plastic yield and repetitive energy dissipation during cyclical strains. Based on these observations, in all subsequent experiments native fibres were subjected to a single conditioning cycle before ion exchange. The highly reproducible mechanical response of conditioned single fibres to cyclical strains allowed baseline parameters to be established for an individual fibre in 1 mM CaCl_2_ solution before evaluating the effects of metal ion exchange on the same fibre. This minimized effects of natural variability from fibre to fibre and animal to animal.

The initial stiffness, yield stress, maximum stress and magnitude of the hysteresis increased following the order: Mg^2+^ < Ca^2+^ < La^3+^ < Zn^2+^ ≈ Fe^2+^ (figure 5*b*,*c*). Mg^2+^ exchange decreased the strength and toughness of the fibres relative to the Ca^2+^ baseline, although reversible yield behaviour was still apparent and recovery of initial length was faster (figure 5*b*). Fibres exchanged with Ca^2+^ changed by 1% or less in all parameters, as expected because native fibres contain mostly Ca^2+^. Exchange of with La^3+^, Zn^2+^ and Fe^2+^, on the other hand, all increased fibre strength and the magnitude of cyclical energy dissipation due to the hysteretic response to cyclical loads (figure 5*c*).

### 3.5. Correlating mechanics with IR spectroscopy

**Figure 6.**
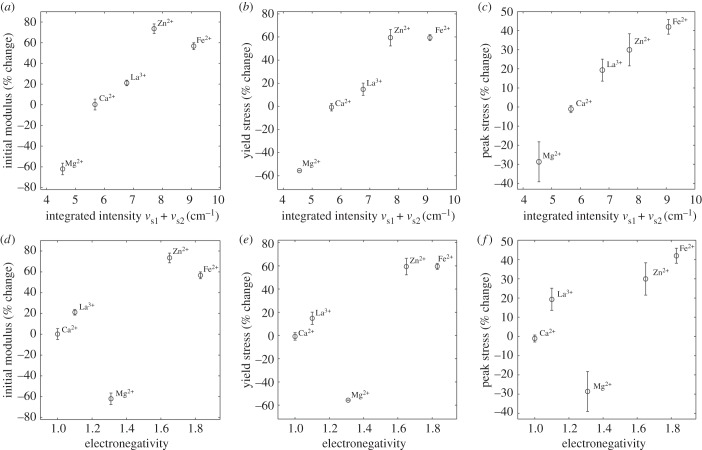
Correlating mechanical properties with phosphate vibrational spectra. (*a*–*c*) Mechanical properties of the silk including initial modulus, yield stress and maximum stress depend on the integrated intensity of the phosphate symmetric stretching modes. (*d*–*f*) The mechanical properties of the silk depend on electronegativity of the metal ion, with the exception of Mg^2+^. Error bars represent the s.d. (*n* = 3). (*d*–*f*).

The effect of the multivalent metal ions on the frequency of the *v*_s_ modes was uncorrelated with their effects on the mechanical properties of the fibres. Therefore, the ionic radius of the metal ions did not determine their mechanical effects on the fibres. On the other hand, the effect of the multivalent metal ions on the integrated intensities of both the *v*_s1_ and the *v*_s2_ bands paralleled their mechanical effect on the fibres (figure 6*a*–*c*). Integrated intensities of the *v*_s1_ (blue peaks, figure 1) and *v*_s2_ modes (green peaks, figure 1) more than doubled, increasing in the order Mg^2+^ < Sr^2+^ < Ba^2+^ < Ca^2+^ < Eu^3+^ < La^3+^ < Zn^2+^ < Fe^2+^. Though the group of metal ions was smaller, the stiffness and strength of the fibres, as reflected in the initial modulus, yield stress and maximum stress, increased in the same order: Mg^2+^ < Ca^2+^ < La^3+^ < Zn^2+^ ≈ Fe^2+^.

## 4. Discussion

The effect of Ca^2+^ on the phosphate IR spectra of caddisworm silk fibres, particularly the splitting of the *v*_s_ band, is atypical of phosphoproteins, though there are only a few studies in the literature for comparison. When Na^+^ was replaced by Ca^2+^ or other multivalent metal ions in the phosphoproteins α_s_-casein [23,24] and hen egg yolk phosvitin [25], the *v*_s_ bands corresponding to the 975 cm^−1^ band of caddisworm silk shifted 10–20 cm^−1^ higher, but a second band *v*_s_ did not appear. These phosphoproteins have metal ion sequestration and storage functions; the metal ions do not have a structural role [26]. Similarly, the –PO_3_ *v*_s_ band of hexaphosphorylated inositol (phytic acid) shifted to 996 from 972 cm^−1^ in Ca^2+^ versus Na^+^ without the appearance of new bands [27]. The *v*_s_ band of the synthetic (pSX)_4_ phosphopeptide also shifted to 995 cm^−1^ from 976 cm^−1^ without the appearance of a new band, which suggested that the unique Ca^2+^-phosphate IR spectra of caddisworm silk (figure 2) is not due only to local sequence effects, like the alternating pattern of phosphoserines, but rather to the higher order structure of [(pSX)_4_]*_n_* domains in the fibres.

The caddisworm silk fibre Ca^2+^/phosphate IR spectra are strikingly similar to the spectra of phosphate compounds in which the phosphates occur in closely spaced, periodic arrangements. In these compounds, the –PO_3_ *v*_s_ band splits into two bands. The numerous examples of *v*_s_ band splitting in periodic phosphate compounds include crystalline phosphate salts (Na_2_HPO_4_·2H_2_O) [28], the phosphate minerals brushite (CaHPO_4_·2H_2_O) [29,30] and dittmarites (e.g. NH_4_MgPO_4_·H_2_O) [31], mononucleotides at high concentration in aqueous solution in which base stacking is thought to bring the phosphate groups into close regular order [32] and in dimyristoylphosphatidic acid lipid bilayers complexed with Ca^2+^ [33]. In the latter case, based on the similarities of the IR spectra to calcium phosphate salts [29], the authors proposed that the decreased 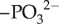 group symmetry and splitting of the stretching mode bands into doublets was due to the periodic arrangement of Ca^2+^-phosphate cross-bridges between lamellar bilayers. In their model, the lipid phosphate groups are fully deprotonated, each of the three P–¨O bonds has a similar partial negative charge (bond order) and each Ca^2+^ ion in the two-dimensional network interacts with four phosphates, two from each of the stacked layers. Their model was consistent with the spacing between bilayers measured by small-angle X-ray diffraction.

**Figure 7.**
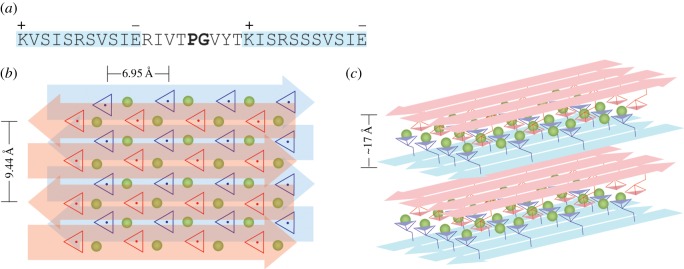
Hypothetical structures of periodic phosphates in caddisworm silk β-domains. (*a*) Primary sequence of a [(pSX)_4_]_2_ motif from the H-fibroin D-repeat. The Lys and Glu may align adjacent anti-parallel strands in a β-configuration as indicated by the + and – symbols. (*b*) Hypothetical arrangement of two anti-parallel [(pSX)_4_]_2_ β-hairpins in a β-sheet (blue arrows) stacked with an identical opposing β-sheet (red arrows) stabilized through Ca^2+^/pS cross-bridging. The green spheres are Ca^2+^ and the red and blue triangles are the orthographic projections of the 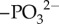 moieties. (*c*) The stacking of several anti-parallel sheets through Ca^2+^ ion (green spheres) cross-bridging of phosphates (triangles) alternating with hydrophobic association of reverse face aliphatic sidechains (not shown).

Based on the dissimilarity of caddisworm silk metal ion-dependent phosphate IR spectra to casein and phosvitin and the similarity to periodic phosphate compounds, we propose that the Ca^2+^ 1007 cm^−1^ band is a *v*_s_ mode that originates from factor group splitting of the Na^+^ 975 cm^−1^ *v*_s_ band due to close proximity and periodic order of Ca^2+^–phosphate complexes in [(pSX)_4_]*_n_* domains. An ordered network of Ca^2+^-cross-bridges between adjacent phosphatidic acid lipid bilayers is structurally similar to the previously proposed Ca^2+^-cross-bridging of two opposing phosphoserine faces of [(pSX)_4_]*_n_* β-sheets in caddisworm silk (figure 7) [7]. A detailed and definitive description of the coordination structure of the Ca^2+^–phosphate complexes in caddisworm silk requires additional experimentation. Nevertheless, an illustrative example of a periodic, two-dimensional arrangement of Ca^2+^–phosphate complexes is presented in figure 7. The two (pSX)_4_ sequences separated by a short sequence with a central PG in the D-repeats of H-fibroin (figure 7*a*) are predicted to form β-hairpins. Lysine (K) and glutamic (E) sidechains, which flank the (pSX)_4_ regions, may stabilize the registry of the anti-parallel β-strands through electrostatic interactions (plus and minus symbols, figure 7*a*). The phosphates are dianionic and in the centre of the sheet; each Ca^2+^ ion interacts with four P–¨O ligands from one β-sheet and two from the opposing β-sheet. The phosphates at the edges of the β-sheets are in a different coordination environment from those in the centre. The model is consistent with previously reported solid-state NMR experiments that demonstrated the phosphoserines are in a β-sheet conformation with limited mobility and are uniformly and fully deprotonated (doubly anionic) [11].

The IR spectra of the other multivalent metal ions are similar to the Ca^2+^ spectra with regard to the number of phosphate bands, indicating exchange with all of the metal ions tested resulted in a similar decrease in the symmetry of the phosphate groups. This suggests that the various multivalent metal ions do not affect the general structure of the [(pSX)_4_]*_n_* β-domains. Variations in the frequencies and integrated intensities of the phosphate stretching modes, on the other hand, indicate subtle structural effects. The integrated band intensity (*Γ*) is related to the transition dipole moment (*δμ*/*δQ*) of the vibrational mode by4.1
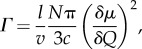
where *v* is the frequency of the vibration, *N* is Avogadro's number, *l* is the path length, *µ* is the dipole moment, *Q* is the transition axis and *c* is the concentration of the absorbing material [34]. Polar bonds with larger transition dipole moments absorb more IR radiation. The integrated intensities of the *v*_s1_ and *v*_s2_ bands increased in the order Mg^2+^ < Sr^2+^ < Ba^2+^ < Ca^2+^ < La^3+^ < Zn^2+^ < Eu^3+^ < Fe^2+^. With a subset of metal ions, the stiffness and strength of the silk fibres increased in the order Mg^2+^ < Ca^2+^ < La^3+^ < Zn^2+^ ≈ Fe^2+^, thereby establishing a direct correlation between the transition dipole moment of the metal ion–phosphate complexes and the mechanical properties of the silk fibres. The greater the electronegativity of the metal ion, the greater the transition dipole moment of the phosphate groups, the stronger the [(pSX)_4_]*_n_* domain cross-bridges, the greater the stiffness and strength of the fibres.

Mg^2+^ is an outlier in the series as its integrated intensities are lower and Mg^2+^ fibres are weaker than Ca^2+^ fibres despite the considerably greater electronegativity of Mg^2+^ versus Ca^2+^. Polyphosphate hydrogels were also mechanically weaker when cross-linked with Mg^2+^ than with Ca^2+^ and other divalent metal ions [3]. An octahedral coordination geometry is preferred by Mg^2+^ ions, which in aqueous solution are strongly coordinated to six water molecules [35,36]. In nucleic acids, Mg^2+^ ions are usually coordinated to outer-sphere phosphate ligands through an intact, or mostly intact, inner-sphere hydration shell [37,38]. The weak Mg^2+^ cross-bridging in the [(pSX)_4_]*_n_* domains, evident in the decreased stiffness and strength of the Mg^2+^-exchanged fibres, may be due to indirect, outer-sphere coordination of Mg(H_2_O)_6_^2+^ ions by phosphate sidechains or as a mix of outer- and inner-sphere coordination with only partial exchange of the hydration shell.

In conclusion, the integrated intensities of phosphate *v*_s_ modes predict fibre stiffness and strength, providing definitive evidence that reversible phosphate/metal ion complexes are responsible for the toughness of caddisworm silk. Furthermore, the phosphate IR spectra have the split *v*_s_ band characteristics of phosphate structures with regular periodic order, evidence the phosphate/metal ion complexes occupy a two- or three-dimensional periodic network, as predicted from the sequence of H-fibroin [6]. In the absence of multivalent metal ions, the Na^+^ fibres are weak progressive springs with a final stiffness nearly the same as the final stiffness of the multivalent metal ion containing fibres (figure 4). The Na^+^ fibres represent the isolated elastic covalent network that provides a restoring force to guide recovery of the Ca^2+^/(pSX)_4_ β-domains during unloading. Caddisworm silk is mechanically well adapted as an underwater structural material. Connecting features of its molecular structure with its toughness and unique mechanical response to strain will reveal further design principles for the development of tough synthetic materials specifically designed for wet environments [3].

## Supplementary Material

Supplemental Material

## Data accessibility

Extended data are provided in the electronic supplementary material (figure S1).
