# Supplementary figures and images for: Connecting caddisworm silk structure and mechanical properties: combined infrared spectroscopy and mechanical analysis
Source: Open Biol. 2016 Jun 8;6(6):160067. doi: 10.1098/rsob.160067 (PMC4929942; doi:10.1098/rsob.160067)

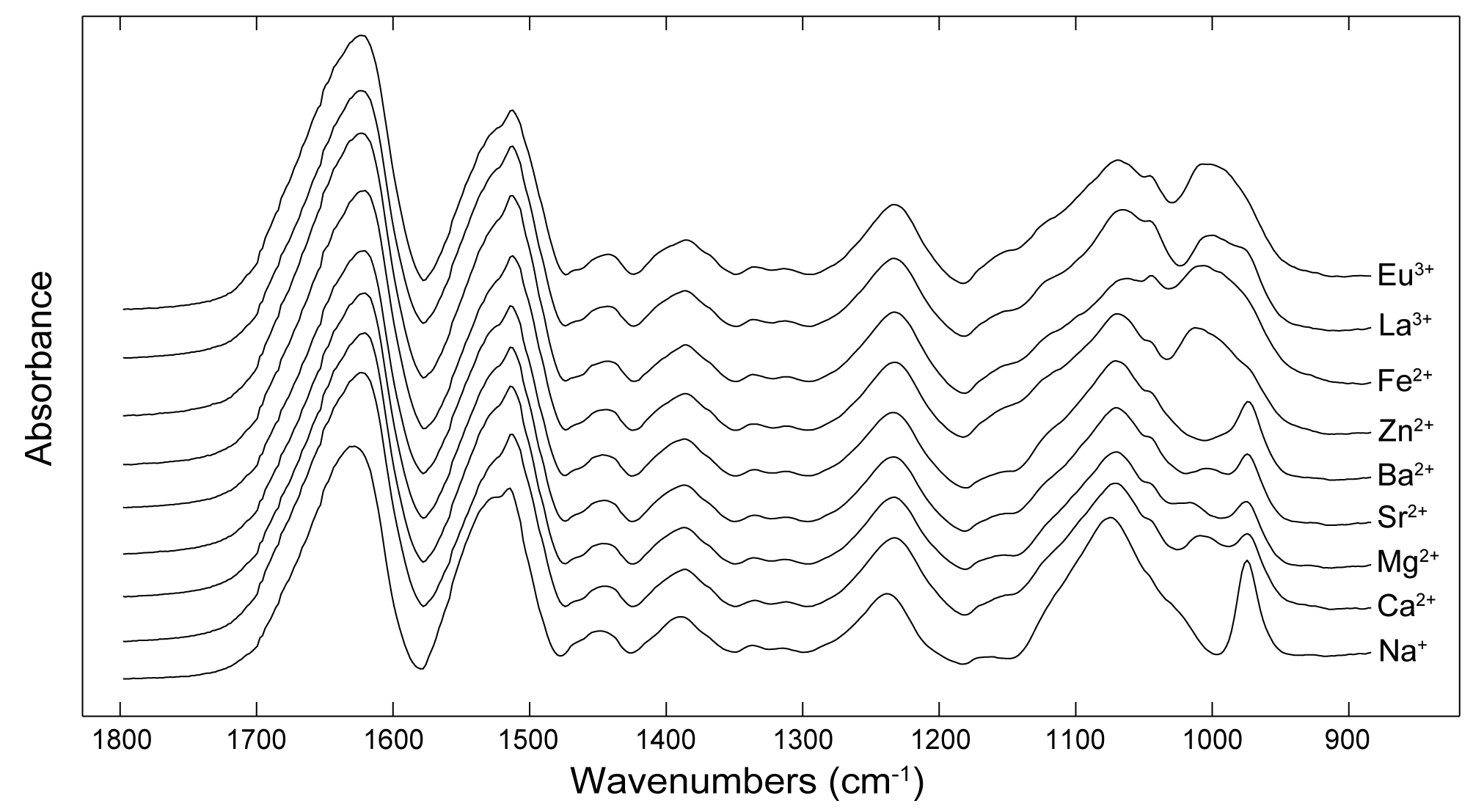

Supplement: Supplemental Material [file rsob160067supp1.tif]
